# DNA damage and metabolic mechanisms of cancer drug resistance

**DOI:** 10.20517/cdr.2021.148

**Published:** 2022-05-05

**Authors:** Deanna Tiek, Shi-Yuan Cheng

**Affiliations:** The Ken & Ruth Davee Department of Neurology, Lou and Jean Malnati Brain Tumor Institute at Northwestern Medicine, Robert H. Lurie Comprehensive Cancer Center, and Simpson Querry Institute for Epigenetics, Northwestern University, Feinberg School of Medicine, 303 E Superior St, Chicago, IL 60611, USA.

**Keywords:** Cancer drug resistance, drug resistance, metabolism, DNA damage, DNA repair, hypoxia, synthetic lethality, overcoming resistance

## Abstract

Cancer drug resistance is one of the main barriers to overcome to ensure durable treatment responses. While many pivotal advances have been made in first combination therapies, then targeted therapies, and now broadening out to immunomodulatory drugs or metabolic targeting compounds, drug resistance is still ultimately universally fatal. In this brief review, we will discuss different strategies that have been used to fight drug resistance from synthetic lethality to tumor microenvironment modulation, focusing on the DNA damage response and tumor metabolism both within tumor cells and their surrounding microenvironment. In this way, with a better understanding of both targetable mutations in combination with the metabolism, smarter drugs may be designed to combat cancer drug resistance.

## INTRODUCTION

Over the past decades, our understanding of cancer as a disease has increased immensely. The realization of using DNA damaging agents to inhibit the growth of fast-dividing cells with chemotherapy was a game-changing step in treating many types of cancers^[1]^. However, as cancer cells are in a more plastic state with increased genomic instability, resistance to single-agent chemotherapy became prevalent. Therefore, following the steps of infectious disease protocols, combination therapies have evolved to combine multiple chemotherapeutic agents to elicit a longer-lasting effect^[2]^. While this is more beneficial than single-agent treatments, drug/therapy resistance in cancer is still inevitable and universally fatal^[3]^.

Targeted therapies have emerged to combat the rapid drug resistance of broad DNA damaging chemotherapy compounds, which use our increased knowledge of specific vulnerabilities in different types of cancers^[4]^. Success has been seen targeting specific proteins, such as BCR-ABL, the estrogen receptor^[5]^, the androgen receptor (AR)^[6]^, HER2^[7]^, the epidermal growth factor (EGFR)^[8]^, and others. More recently, targeting the immune system via checkpoint inhibitors, like PD1/PD-L1^[9]^ and CTLA4^[10]^, have produced cures in a subset of patients. Nevertheless, in patients where a cure cannot be achieved with targeted or conventional chemotherapy, cancers will recur and become drug-resistant which ultimately leads to patients’ death. Therefore, drug resistance is the principal limiting factor in patient overall survival.

In this review, we discuss relevant resistance mechanisms tumor cells use to adapt to both chemotherapy and targeted therapies. Furthermore, we summarize some of the promising avenues that are currently being investigated to target the tumor resistance pathways and mutations that arise from the treatments. Overall, a better mechanistic insight into drug-resistant cells will hopefully allow for smarter drug design to help combat the major problem of drug resistance and extend patient survival.

## MECHANISMS OF RESISTANCE

### Changes in the drug-induced DNA damage response

DNA damage continues to be an effective target for cancer therapy as the definition of cancer is uncontrolled cell growth^[4]^. In this way, increased cell cycling can lead to more error-prone DNA replication, which relies on DNA damage repair pathways to ensure cell fitness. Therefore, targeting DNA replication via chemo- and radio-therapy to induce DNA damage and ultimately cell death is still the most common - and sometimes the most effective - in cancer treatment^[11]^. However, while chemo- and radio-therapy can be initially successful, therapy resistance in cancer is common and ultimately fatal. For this reason, extensive efforts have been focused on both determining and targeting the protein or pathways involved in chemo- and radio-resistance.

In glioblastoma (GBM) - an extremely deadly brain cancer with a ~14-16-month median survival rate - the standard of care includes radiation therapy (RT), maximal surgical resection, and the chemotherapeutic agent temozolomide (TMZ)^[12]^. The mechanism of action of TMZ was later shown to create O^6^-methylguanine adducts, which would create double-strand breaks (DSBs) post replication. However, TMZ-resistance is rapid and was found to be partly due to the DNA damage repair protein O^6^-methylguanine-DNA methyltransferase (MGMT)^[13]^. MGMT is the suicide DNA repair protein responsible for removing the O^6^-methylguanine adducts and allowing for its damage repair over DSB formation and cell death^[14]^. In this way, MGMT inhibitors have been reported that inhibit the function of MGMT. MGMT inhibition has also been shown, to reverse pancreatic tumor gemcitabine resistance via suppressing the expression of survivin in animal models^[15]^. However, little clinical success has been realized with MGMT inhibitors with or without TMZ treatment^[16]^.

DNA-protein kinase (DNA-PK) is a DSB DNA damage sensing complex composed of Ku70, Ku80, and the DNA protein kinase catalytic subunit (DNA-PKcs)^[17]^. After a DSB has occurred, DNA-PK binds to the broken end of the DSB to protect it from nuclease degradation and recruits the other DNA damage repair proteins to initiate DSB repair via non-homologous end joining (NHEJ)^[18]^. RT has been shown to induce DSBs of which DNA-PK can identify and repair with NHEJ, thereby preventing radiation-induced cell death^[19]^. Cells which have a decreased expression of DNA-PKcs have also been shown to be more sensitive to RT^[20]^. In this way, many DNA-PKcs inhibitors have been developed to overcome radiation resistance and/or enhance radiation-induced cell death. Radio-sensitization via DNA-PKcs inhibition has been observed with DNA-PK inhibitor VX-984 in GBM^[21]^, NU7441 in cervical and breast cancers^[22]^, and NU7026 in non-small cell lung cancer^[23]^. Another DNA-PK inhibitor, NU5455, has also been shown to enhance the killing of doxorubicin in lung cancer models^[24]^.

Lastly, it has been shown that chemotherapy can modulate pro-survival pathways like increasing the expression of drug efflux pumps, apoptosis defects, DNA adduct tolerance, cellular detoxification, and inducing a hypoxic environment^[25]^. While the metabolic effects of hypoxia will be discussed in more detail below, Chen *et al.*^[26]^ have taken advantage of intracellular hypoxia in cisplatin-resistant cells and created a hypoxia-amplifying DNA repair-inhibiting (HYDRI) nanomedicine. HYDRI specifically targeted cancer cells because of their drug-induced hypoxic environment, and then released its payload of hypoxia-activatable chemotherapeutic tirapazamine. In this way, previous studies which determined upregulated pathways in cisplatin resistance could be used to create a smarter therapy that bypassed drug efflux pumps, induced a unique DNA damage profile, and relied on the inevitable hypoxic environment created by cisplatin resistance to target these drug-refractory models^[26]^.

### Synthetic lethality

**Figure 1:**
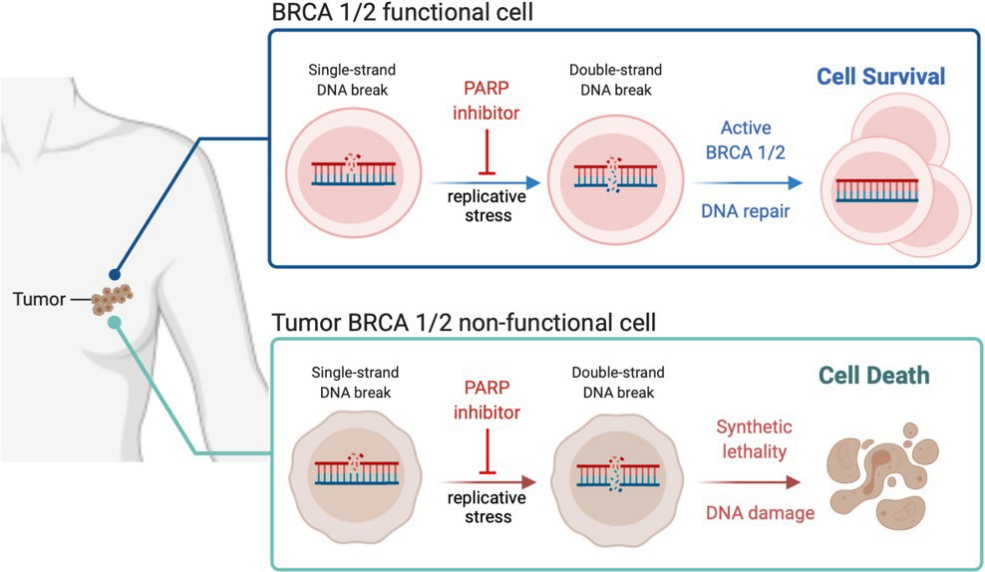
PARPi synthetic lethality in BRCA 1/2 non-functional tumors. BRCA 1/2 functional tumor cells will repair the double-strand break (DSB) induced by PARP inhibition and sequential replicative stress allowing cell survival and growth, whereas BRCA 1/2 non-functional cells cannot repair the DSB and therefore succumb to DNA damage-induced cell death. Figure created with BioRender.

In 2005, the idea of synthetic lethality in the DNA damage repair pathway, via BRCA1 mutation, was published by two groups^[27,28]^. Poly ADP ribose polymerase (PARP) 1 is a key activating protein in the single-strand break (SSB) or base excision repair DNA damage repair pathway^[29]^. In parallel, a double-strand break (DSB) can be fixed by two major DNA damage repair pathways - homologous recombination (HR) or NHEJ. HR utilizes a sister chromatid and has a lower error rate, with BRCA1/2 playing a major activating role for proper DNA damage repair, whereas NHEJ is more error-prone, but quicker in repairing a DSB in interphase^[30]^. Upon the advent of genetic profiling of tumors, it was discovered that many breast and ovarian cancers had either germline or tumor-specific mutations in the *BRCA1* or *BRCA2* gene^[27]^. With the knowledge that SSBs that were unresolved by PARP1 became DSBs with replicative stress that requires BRCA1/2 for repair, the idea of cancer DNA damage response (DDR) synthetic lethality was tested^[31] ^[Figure 1]. PARP1 inhibition (PARPi) in BRCA1/2 mutated tumors has been successful in many types of cancers, including breast, ovarian, prostate, pancreatic, colon, and lung^[32]^. One of the main advantages of tumor specific BRCA mutations is the decreased toxicity of single-agent PARPi treatment. Combination therapies to mimic this synthetic lethality by combining both a BRCA inhibitor with PARPi have proven to be toxic^[33]^.

Currently, there are at least five PARPi - veliparib (Abbvie), rucaparib (Pfizer/Clovis), Olaparib (KuDOS/AstraZeneca), niraparib (Merck/Tesaro), and talazoparib (Lead/Biomarin/Medivation/Pfizer)^[32]^ - where the most common mechanism of action is the “trapping” of PARP on the DNA to induce DSBs^[34]^. Talazoparib is the newest PARPi and has the highest ability to “trap” PARP on the DNA with ~100 times greater efficacy than the next best PARPi^[35]^. Nevertheless, this increased trapping of PARP increases the toxicity of talazoparib - compared to other PARPi^[36]^. In clinical results, the phase 3 trial EMBARCA had 431 BRCA1/2 mutant patients with advanced breast cancer, where the talazoparib group had a 62.6% response rate compared to the 27.2% of the standard chemotherapy group^[37]^.

The synthetic lethality success story has other groups actively looking for other synthetic lethal interactions with the many abundant cancer-associated mutations. While not dependent on DDR mutations, AR signaling and PARPi have also been shown to give rise to a synthetic lethal phenotype in preclinical models. However, it is important to note that 19% of primary prostate cancers and 23% of metastatic castration-resistant prostate cancer (mCRPC) have DNA damage repair gene mutations. BRCA2 mutations also have increased levels of prostate specific antigen, a larger percentage of high Gleason scored tumors, and elevated rates of distant and nodal metastases^[38]^. Clinically, prostate cancer patients treated with abiraterone plus olaparib showed improved radiographic progression free survival over abiraterone alone in a phase 2 trials which was independent of DDR mutations^[39]^. This has also led to an interest in combining PARPi with androgen deprivation therapy alone or in combination with AR signaling inhibitors, which is currently ongoing in mCRPC, and should be considered even in a non-DDR altered state^[38]^.

### DNA damage response with immunotherapy

Immune checkpoint inhibitors (ICI) had great initial promise, with early clinical trial results showing obvious tumor shrinkage, initially. However, after further evaluation, ICI can have about a ~10%-20% durable response rate, depending on the types of cancer^[40]^. Therefore, like what we previously described with chemotherapy and targeted therapy, combinatorial studies have been designed to determine whether ICI efficacy can be improved when combined with other conventional therapies^[41,42]^.

Melanoma was the first cancer to show preliminary success with ICI, and it is well-known that melanoma has one of the highest rates of tumor mutation burden (TMB)^[43]^. This brought about the hypothesis that higher rates of TMB in cancer would increase the number of neo-antigens which were predicted to produce a stronger immune response and increase sensitivity to ICI. Radiation and ICI have been tested in combination as radiation treatment for cancer will induce DNA damage, neo-antigens, and immune response^[44]^. Furthermore, it was shown in 1979 that the effect of radiation is linked to the immune system when twice the dose of radiation was needed to control tumor growth in thymectomized mice compared to mice with an intact immune system^[45]^. Within the clinic, overall survival of concurrent radiation and ICI - compared to radiation before or after ICI - was shown to improve overall survival (OS) in a retrospective review of lung cancer patients with distant brain metastases^[46]^.

Other chemotherapy agents are currently being combined with ICI to determine combinatorial efficacy, where one combination - PARPi + ICI - is the most developed. PARPi have shown great promise in many avenues, as shown above with synthetic lethality strategies^[47]^. With the increasing characterization of PARPi pathway changes, it was noted in breast cancer that PARPi induced PD-L1 expression^[48]^. Not only has PD-L1 been shown to increase with PARPi, but also an increase in cytoplasmic DNA, which activates the cyclic guanosine monophosphate-AMP synthase (cGAS)/stimulator of interferon genes (STING) pathway^[47]^. PARPi has also been shown to inactivate the glycogen synthase kinase 2 beta (GSK3)^[36]^. An *in vivo* model of BRCA-deficient triple-negative breast cancer also demonstrated that PARPi activated the cGAS/STING pathway and increased CD8^+^ T cell infiltration^[48]^, as well as decreasing T-cell activation resulting in enhanced cancer cell apoptosis^[36]^. Currently, biomarkers are being investigated to select non-BRCA patients that would respond to PARPi + ICI, where the mutational signature 3 - associated with HR deficiency - positively predicted patient responses^[49]^. Both the TOPACIO trial and MEDIOLA study are investigating feasibility of immune checkpoint blockade with PARPi^[36]^.

### Reactive oxygen species in drug resistance

Reactive oxygen species (ROS) play a well-known role in cell growth and proliferation in cancer cells, where an increase in ROS can enhance cell growth and post-treatment survival^[50]^. One way in which ROS can increase cancer cell survival is genetically by oxidizing nucleic acids, which will cause random mutations and increase genomic instability^[51,52]^. ROS can also affect the normal redox balance within the cell. Cysteine is a readily oxidizable amino acid containing a thiol (-SH) group^[53]^. Many enzymes have active sites that contain necessary cysteine residues to assist in biochemical reactions^[54]^. In Fms-related receptor tyrosine kinase (FLT)3-ITD (a mutation in the tyrosine kinase domain) expressing acute myeloid leukemia, NADPH oxidase 4 generated ROS will inactivate the protein-tyrosine phosphatase (PTP) DEP-1/PTPRJ, which negatively regulates FLT3-ITD transformation^[55]^. PTP phosphatase and tensin homolog (PTEN), a PTP family member and potent tumor suppressor, has also been shown to be susceptible to H_2_O_2_-mediated oxidation and inactivation^[56]^. As PTEN is a negative regulator of PI3K and Akt pathways, oxidation and inactivation of PTEN augments downstream signaling and cell growth^[57]^.

While ROS have been used to create DNA damage via chemotherapy and are necessary byproducts of radiation, drug-resistant cells have been shown to increase their intracellular ROS levels, and thereby adapt to this intracellular hypoxic environment^[58]^. Initially, when naïve cells are exposed to chemotherapy, an increase in ROS is noted, as well as a concomitant increase in the antioxidant systems to combat this onslaught of oxidants^[58]^. However, this seems to differ in some drug-resistant, or persister, cells where an increase in ROS is still true, but antioxidant genes like glutathione peroxidase (GPX) 4 are now downregulated^[59]^. One hypothesis is that these cells use this ROS to their advantage as the hypoxia response element (HRE) shows a higher binding of HIF1 when the G’s of the HRE are modified via ROS^[60]^. Accordingly, in a new study which dives into the metabolic and transcriptional changes between untreated and persister cells post drug treatment, two of the main upregulated pathways in their model, osimertinib-treated Trp53-knockout with a lung-specific EGFR (L858R) mutation, were ROS and fatty acid metabolism (FAM)^[61]^. Furthermore, the cells that had the increased ROS/FAM gene signature were also the cycling persister cells, compared with the non-cycling persister cells. In post-treatment patient samples, 8 out of 11 melanoma samples had an increase in either ROS or FAM signatures, as well as 50% of HER2^+^ breast cancer samples, but the increase in HER2^+^ samples was only in the post-treatment samples^[61]^.

In these ways, ROS-induced metabolic reprogramming has been an active non-mutational target. The most promising area of research is ferroptosis - or iron-dependent cell death - induction^[62]^. As drug-resistant cells have been shown to increase ROS and decrease their antioxidant gene expression, this leaves the cells exquisitely sensitive to ferroptosis via GPX4 or xCT inhibition^[59]^. GPX4 is the main regulator to decrease lipid oxidation, and xCT is a cystine/glutamate antiporter, where cysteine is a necessary component for the reducing agent glutathione^[63]^. While ferroptosis induction has shown promising initial results in drug-resistant cell and animal models, most drugs never reach the target in cancers such as pancreatic cancer. In this largely drug-refractory cancer, Badgley *et al.*^[64]^ showed that a cyst(e)ine degrading enzyme - cyst(e)inase - was able to deplete cyst(e)ine from the extracellular environment, thereby decreasing intracellular cyst(e)ine and preventing GSH production. In both *in vitro* and *in vivo* models, cyst(e)ine deprivation led to robust induction of ferroptosis, cell death, and longer animal survivals^[64]^.

### Drug resistance via lipid metabolism and import

Fatty acid (FA) metabolism plays a host of roles within the cell. FAs may be most well-known for being membrane building blocks with the synthesis of glycerophospholipids^[65]^. Interestingly, the lipid composition of membranes has garnered recent interest as chemotherapy-resistant cancer cells, in preclinical models, show reduced fluidity of their membranes. These membranes have an increase in saturated fatty acyl chains and are especially enriched for monounsaturated fatty acyl chains in glycerophospholipids^[66]^. While this may seem inconsequential, one of the most promising therapeutic targets of drug-resistant cells - ferroptosis - can depend on poly-unsaturated fatty acyl chains to induce toxic lipid peroxidation and cell death^[67]^. Furthermore, in chemotherapy-resistant leukemia or ovarian cancer cell lines, the reduced membrane fluidity can come from an increase of cholesterol and/or sphingomyelin within the membrane^[68,69]^. This stiffened membrane has been shown to decrease passive diffusion of drug uptake and enhance detergent-resistant membrane domains, which can activate the family of ATP-binding cassette multidrug efflux transporters - including p-glycoprotein - potentiating the multidrug-resistant (MDR) phenotype. However, modulation of membrane fluidity was able to alter the drug efflux transporters, suggesting a potential for diet interventions^[70]^.

Targeting lipid synthesis itself may also have a benefit in re-sensitizing cells to chemotherapy. Fatty acid synthase (FAS) inhibitors have re-sensitized ovarian cells *in vitro*^[71,72]^, *ex vivo*^[71]^, and *in vivo* for T cell lymphoma and ovarian cancer models^[73,74]^. In breast cancer cells, overexpression of FAS was able to confer chemoresistance *in vitro*^[72]^. While the mechanism by which FAS inhibition can alter chemotherapy sensitivity is unknown, a decrease in MDR proteins has been observed, suggesting membrane composition may be important^[75]^. Fatty acid oxidation has also been shown to increase with chemotherapy resistance. GBM cellular and patient-derived xenograft models showed an increase in fatty acid beta-oxidation post-TMZ treatment^[76]^. In breast cancer patient samples, the necessary beta-oxidation enzyme carnitine palmitoyltransferase I (CPT1) was increased in tumors that recurred and was also higher in chemo-resistant tumors^[77]^. CPT1 inhibitors have also been shown to re-sensitize tumor cells to chemotherapeutic agents^[78]^.

**Figure 2:**
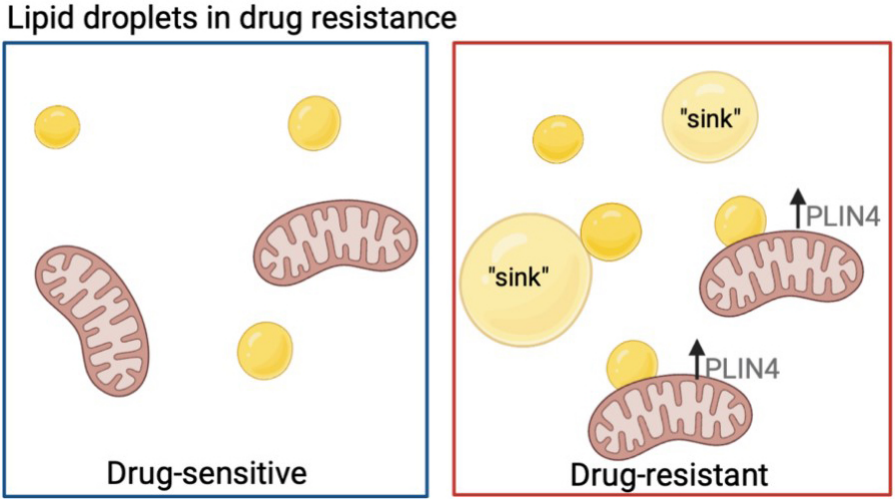
Lipid droplet usage in drug-resistant cells. Drug-resistant cells have been shown to increase lipid droplet accumulation (yellow circles) and have a higher percentage co-localized to the mitochondria, where an increase in PLIN4 helps to better utilize lipids for fatty acid beta-oxidation. Lipids can also be used as drug “sinks” for hydrophobic drugs. Figure created with BioRender.

Lastly, lipid droplet (LD) number and function play an important role in chemotherapy resistance. LDs may directly assist in cell survival by providing an energy reserve of lipids to be oxidized in case of nutrient deprivation^[79]^. Hydrophobic drugs can also be sequestered within lipid droplets, creating a drug “sink” for detoxifying chemotherapeutic agents^[79]^. Interestingly, LDs were found to co-localize with the mitochondria more frequently in chemo-resistant breast cancer cells, where the LD protein perilipin 4 (PLIN4) was increased. PLIN4 assists in mobilizing lipids for oxidation from LDs, where silencing of PLIN4 decreased the growth of the chemotherapy-resistant, but not the parental, breast cancer cells, suggesting that lipid beta-oxidation is necessary for the sustained growth of chemotherapy-resistant cells^[80] ^[Figure 2]. Inhibition of long-chain fatty acyl-CoA synthetase via triacsin C blocked fatty acid activation and LD biogenesis which rendered drug-resistant colorectal cancer cells sensitive to chemotherapy treatment both *in vitro* and *in vivo*^[81]^.

### Hypoxia and drug resistance

The 2019 Nobel Prize was awarded to Kaelin, Ratcliffe, and Semenza for their seminal work on discovering how oxygen is sensed in the cell and the way in which cells are able to adapt to changing oxygen concentrations^[82,83]^. Hypoxia-inducible factor (HIF) and HIF signaling are now realized as both a hallmark of cancer, and also affect the surrounding microenvironment^[4]^. Through large data analyses, Bhandari *et al.*^[84]^ used the Buffa-defined hypoxia signature to determine the breadth of hypoxia amongst 1188 samples from 27 types of cancer. They found that hypoxia was varied both between cancers and even within a single patient. The most hypoxic tumors were cervical squamous cell carcinoma and lung cancer, with thyroid adenocarcinoma and chronic lymphocytic leukemia being the least hypoxic^[84]^. This study corroborated the findings of The Cancer Genome Atlas. Furthermore, higher hypoxia scores also correlate to both lower overall survival and progression-free survival in multiple cancer types^[85]^. A forced hypoxic environment in GBM models has also been shown to decrease the sensitivity of cells to the standard of care chemotherapeutic agent TMZ^[86]^.

As cancer cells grow faster than normal cells, they quickly outgrow their nutrient supply, which creates a lower level of oxygen in the tumor microenvironment (TME). This low oxygen, or hypoxic TME, affects gene signatures and pathways activated within the tumor cells^[87]^. Carbonic anhydrases and CO_2_ levels are increased, which leads to increased cellular acidification^[88]^. However, cancer cells depend on a higher intracellular pH (~7.4 *vs.* ~7.2) and acidify their TME, decreasing the extracellular pH (~6.0-~7.1 *vs.* ~7.4)^[89]^. This acidic niche has been shown to increase the expression of MDR genes and decrease drug import into cancer cells^[90]^. A highly acidic TME can also prevent proper immune profusion and cause resistance to ICI^[91]^. Acidity can increase the immune checkpoint protein expression, CTLA-4 on T cells, raise the threshold for T cell activation^[92]^, and decrease CD8^+^ memory T cell lifespan^[93]^. Long-term exposure to an acidic environment can decrease natural killer cell function, activation, and survival^[92]^. Therefore, modulation of TME pH has been an active area of research.

To neutralize the acidic TME, oral bicarbonate was given and artificially increased the pH of the TME, which allowed for a better response to ICI in multiple cancer models^[94]^. In an effort to maintain normal levels of pH, Na^+^/H^+^ exchanges, like sodium-hydrogen exchanger isoform (NHE), are upregulated to uptake sodium and pump out protons^[95]^. The Na^+^/H^+^ exchangers are the most common membrane proteins and attempt to regulate the hypoxia-induced pH changes within the cell^[96]^. As intracellular acidosis can induce necroptosis and apoptosis, NHE1 inhibitors have been found to modulate intracellular pH and lead to cell death^[97]^. Cariporide, an NHE1 inhibitor, can induce apoptosis in breast cancer, reduce MDR1 expression, and decrease tumor volume^[98]^. Another NHE inhibitor, amiloride, increased ROS abundance, thereby stimulating PAR synthesis and inducing the PAR-dependent cell death termed parthanatos^[99]^. In GBM, Na^+^ was shown to be increased almost 3-fold between cancer and normal cells, while NHE1 overexpression is noted and increased NHE1 correlated to worse overall survival^[97]^.

## CONCLUSIONS AND FUTURE PERSPECTIVES

Cancer drug resistance remains the biggest challenge in successfully treating cancer patients today. Here we have outlined some of the current strategies to target the DNA damage repair proteins via chemo-induced dependencies, synthetic lethality, and combination with immunotherapy. More research insight into cancer-specific deficiencies can only lead to better responses, or third-line therapeutic options. Tumor metabolism and the microenvironment may also prove to be promising drug targets as uncontrolled cell growth will always be a hallmark of cancer. Therefore, if we are truly able to understand the metabolic changes and vulnerabilities of cancer, we may be able to develop biomarkers to help dictate metabolic status and treatment plans.

**Figure 3:**
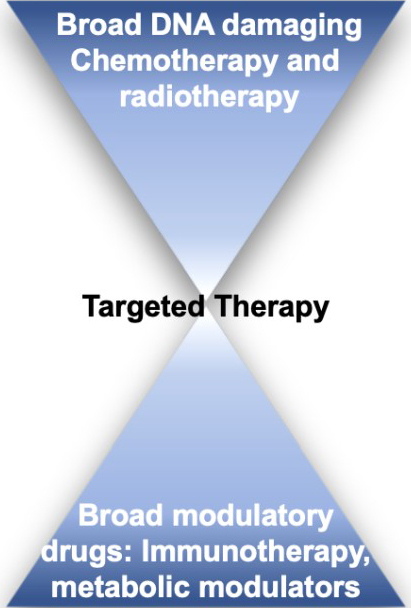
Therapy schematic. Broad-reaching drugs may lend to more durable responses as resistance can arise more rapidly to targeted therapy. Modulating more broad cancer hallmarks - like immune and metabolic targets - may offer smarter drug targets.

As a whole, we may be better off looking at broad regulatory pathways, as we seem to be at the far end of an hourglass curve [Figure 3]. In the beginning, broad DNA damaging agents were game-changing as they targeted the quintessential cancer dependency - cell growth. Then we narrowed the focus to specific proteins with either activating mutations or cancer-driving functions. While some success has been achieved with targeted therapy, new avenues like modulating the immune system, or using HYDRI-like methods to target broad cancer hallmarks may lead to more durable and smart therapeutic designs.

## DECLARATIONS

### Copyright

© The Author(s) 2022.
